# A computational model of stem cells’ internal mechanism to recapitulate spatial patterning and maintain the self-organized pattern in the homeostasis state

**DOI:** 10.1038/s41598-024-51386-z

**Published:** 2024-01-17

**Authors:** Najme Khorasani, Mehdi Sadeghi

**Affiliations:** 1 School of Biological Sciences, Institute for Research in Fundamental Sciences (IPM), Tehran, Iran; 2 National Institute of Genetic Engineering and Biotechnology (NIGEB), Tehran, Iran

**Keywords:** Computational models, Nonlinear dynamics, Multistability, Extracellular signalling molecules, Self-renewal, Stem-cell differentiation

## Abstract

The complex functioning of multi-cellular tissue development relies on proper cell production rates to replace dead or differentiated specialized cells. Stem cells are critical for tissue development and maintenance, as they produce specialized cells to meet the tissues’ demands. In this study, we propose a computational model to investigate the stem cell’s mechanism, which generates the appropriate proportion of specialized cells, and distributes them to their correct position to form and maintain the organized structure in the population through intercellular reactions. Our computational model focuses on early development, where the populations overall behavior is determined by stem cells and signaling molecules. The model does not include complicated factors such as movement of specialized cells or outside signaling sources. The results indicate that in our model, the stem cells can organize the population into a desired spatial pattern, which demonstrates their ability to self-organize as long as the corresponding leading signal is present. We also investigate the impact of stochasticity, which provides desired non-genetic diversity; however, it can also break the proper boundaries of the desired spatial pattern. We further examine the role of the death rate in maintaining the system’s steady state. Overall, our study sheds light on the strategies employed by stem cells to organize specialized cells and maintain proper functionality. Our findings provide insight into the complex mechanisms involved in tissue development and maintenance, which could lead to new approaches in regenerative medicine and tissue engineering.

## Introduction

Transition from unicellularity into multicellularity occurs through irreversible differentiation, and self-organization. Multicellularity makes complex functionalities possible through inter-cellular communication, and cooperation between phenotypically different cell types of a tissue. Through differentiation the right proportion of genetically homogeneous specialized cells are produced, the required cells for proper functionality of a living tissue. Through self-organization, as one of the main multicellularity-specific processes, specialized cells are organized to form the desired structure of the tissue corresponding to its specific functionality. In a fully formed organism, the proper functioning of all developed tissues makes life possible.

Stem cells (SCs) play a leading role in both the development and maturation of tissue. It can be inferred that it is essential to study the internal mechanisms of stem cells. This is because they orchestrate their critical decisions in order to keep living systems alive. To fulfill this, we need to develop a mathematical model to recapitulate the stem cells’ internal mechanisms in order to: first, regulate the required non-genetic diversity in the tissue by orchestrating proliferation and differentiation in stem cells, second, organize the population of cells in a desired spatial arrangement, and third, maintain the structure of the population in the dynamic stochastic environment of a living tissue^1^. This will help us to understand mechanistically the critical building blocks of living systems, stem cells, to identify components that can be modified through stem-cell-based therapies to control mysterious phenomena such as aging, and cancer, and generate spatially ordered tissues for therapeutic purposes^2,3^.

Prior research underscores the importance of population heterogeneity arising from genetically similar stem cells^2,3^. This heterogeneity is critical for maintaining a balance between proliferation and differentiation, ensuring the continued existence of the main source of population (SCs) as well as specialized cells with vital role in preserving the tissue’s functionality. To address the first goal of regulating non-genetic diversity, we acknowledge the crucial role of controlled stochasticity in generating population heterogeneity^3–9^. Therefore, our model systematically integrates stochasticity at every level, aiming to faithfully capture the intricate dynamics observed in living tissues. Besides, regulatory networks have been studied as the main decision-makers in a wide range of biological systems^10–22^ and in the presence of stochasticity^4,6,11,17,23–27^. Therefore, a multi-stable regulatory network in the form of a set of ordinary differential equations (ODEs) is considered as the decision-making unit for stem cells in our model^2,3,20,28^. Traditionally, the Gillespie algorithm is considered to be a “golden standard” for describing the behavior of systems with a small number of determinants driven by inherent fluctuations^3,6,29^ without having to deal with complex mathematical equations. By considering probabilities as an integral part of any living system, here we used the Gillespie algorithm to simulate the time evolution of our designed stochastic system.

The regulatory networks orchestrate proliferation/differentiation balance to provide population heterogeneity, and maintain a homeostasis state^3^. To address the second and third objectives, we acknowledge that intercellular communication stands out as the primary factor in establishing and maintaining the structure of the population^9,30^. Consequently, our model is enriched with an intercellular communication mechanism. This means that the decision-making process of stem cells is influenced not only by intrinsic factors but also by extracellular signals capable of diffusing among population cells^3,31^. To achieve this, a reaction-diffusion process is incorporated into the model, facilitating the formation of a spatial pattern within the population and its subsequent maintenance in a steady state.

Focusing on the impact of stochasticity, the proposed model is defined based on six basic principles discussed in previous studies^2,3,9^ as follows: (i) stochasticity is an inevitable part of any living cell^2,8,9,26,32–36^. (ii) two major sources of stochasticity, the non-deterministic position of the cell division plane and nonuniform distribution of determinants in the cell lead to the random distribution of the cytoplasmic molecules among daughter cells during cell division^2,9,37–43^. (iii) cell fate is determined based on the number of determinants in the offspring upon the completion of cell division and assumed to be fixed during cell life cycle^2,9^. (iv) cell determinants interact with each other via an internal switch^44,45^. (v) the decision bias in the internal switch is determined by model parameters representing interactions between the switch elements^2,9^. (vi) the switch parameters could also be affected by the cell location in its environment, and it is the key to the spatial pattern in the population^9^.

In this project, we propose a computational model to describe the stem cells’ internal mechanism leading to self-organization by considering signal diffusion in cell-cell communication, and stochasticity at all levels of simulations. This project demonstrated the ability of our designed model to function as a stem cell mechanism underlying the formation and maintenance of desired spatial patterns in the population (fitted to population functioning). Besides, in the presence of controlled noise our model could easily reach the required population heterogeneity and homeostasis state. Our previous model was modified since it was able to manipulate cellular decision-making biases in order to allow daughter cells to be born in their destined territory, then defend it. Furthermore, we show that the desired spatial order of cells in the population could be determined by a “leading” initial signal corresponding to that desired pattern. In addition, we discuss the impact of the death rate as a key factor in our system. Finally, to further illustrate the strength of the model, we explore the behavior of the system in the absence of one of its constraints, the dish wall.

## Methods

### Mathematical model of the system

We consider building on our previously designed model^3^ to study the capacity of that to imitate the behavior of human embryonic stem cells (hESCs) in early development. To be specific, we aim to model the production and rearrangement process through which a mass of homogeneous cells, as the initial state of the system, gives rise to a desired spatially ordered sequence^46^ as the final state of the system. A spatially ordered population of cells determines the final state of a system, which consists of stem cells (*S*), two specialized cells (*A*, and *B*) and progenitor cells (*P*) as intermediate cells. It is worth mentioning that the presence of progenitor cells prevents the accumulation of mutations in the stem cells, by taking the responsibility of producing specialized cells through a large number of cell division cycles^3^. The stem cells are capable of self-renewing and/or differentiating into progenitor cells, and they appear to be a bi-stable system. Progenitor cells, on the other hand, can self-renew and/or give rise to two non-dividing terminally-differentiated cells termed *A*, and *B*^3,46^. Like the previous version of the model, the progenitor cells are considered as a tri-stable dynamical system with a limited capacity of proliferation and restricted potential of differentiation^47^. To be able to study the potential reactions in the model we should emphasize the existence of three phases in the system: the first phase which is demonstrated by a homogeneous mass of stem cells, the second phase which starts with the first division of stem cells and ends with a population of stem cells, progenitor cells, and differentiated cells in a desired spatially ordered pattern, and the third phase which begins with pattern formation, as the final state of the second phase, and terminates to the tissue homeostasis state.

Pattern formation could only happen in response to the signaling pathways^30^. As BMP4 breaks symmetry in inner cell mass during development, our model also requires a signal, referred to as the “leading” signal, which performs the same function. As we prefer to challenge the model to be able to control the overall population behavior without any outside controller, we assume that in the first, and second phases of the simulation, population cells secrete the primary signal needed to form the desired ordered array of cells in the final state. We are aware of the fact that in early development, pattern formation is much more complex and occurs along a hierarchical path. A first signal establishes the foundation for initial symmetry breaking. This leads to the birth of distinct differentiated cells, and then the presence of distinct signaling pathways. These pathways also could lead to producing even more differentiated cells by breaking the symmetry in their corresponding territory. Finally in three weeks the complex structure of Grastula emerges as a result of a finite loop of secreting a signal and breaking the existing symmetry^30,46^. However, in this model, we skip the hierarchical aspect, and the final pattern is formed in one step. A model that can establish and maintain a desired population pattern in two phases could be utilized by the new-born cells in the population, in order to facilitate the above-mentioned hierarchical process.

In the first phase, stem cells do not divide. However, they are involved in the process of producing the required “leading” signal. When the division of the population is triggered in the second phase of the simulation, progenitor cells (*P*) emerge from the population. Subsequently, differentiated cells (*A* and *B*) are born and finally formed in a spatial pattern corresponding to the “leading” signal. In the third phase, the system reaches its stable steady state and maintains it.

Because of the remarkable degree of flexibility that reversible transfer of cells could provide in the case of injury, and even under normal conditions, here, we believe that stem cells can transit to progenitor cells at a fixed rate and vice versa^3,48–58^. Within the described system, the dynamics of the model are described as follows:

1 $$\begin{aligned}{} & {} \hbox {S}\mathop {\longrightarrow }\limits ^{\eta }\hbox {S} + \hbox {P}, \hbox {S}\mathop {\longrightarrow }\limits ^{\eta _{S}}\hbox {S} + \hbox {S}, \hbox {S}\mathop {\longrightarrow }\limits ^{\eta _{P}}\hbox {P} + \hbox {P}. \end{aligned}$$

2 $$\begin{aligned}{} & {} \hbox {S}\mathop {\leftrightharpoons }\limits ^{w_{P}}_{w_{s}} \hbox {P}. \end{aligned}$$

3 $$\begin{aligned}{} & {} \hbox {P}\mathop {\longrightarrow }\limits ^{\lambda _{P}}\hbox {P}+\hbox {P}, \hbox {P}\mathop {\longrightarrow }\limits ^{\lambda _{A}}\hbox {A}+\hbox {P}, \hbox {P}\mathop {\longrightarrow }\limits ^{\lambda _{B}}\hbox {B}+\hbox {P},\nonumber \\{} & {} \hbox {P}\mathop {\longrightarrow }\limits ^{\mu _{d}}\hbox {A}+\hbox {B}, \hbox {P}\mathop {\longrightarrow }\limits ^{\mu _{A}}\hbox {A}+\hbox {A}, \hbox {P}\mathop {\longrightarrow }\limits ^{\mu _{B}}\hbox {B}+\hbox {B}. \end{aligned}$$

4 $$\begin{aligned}{} & {} \hbox {A}\mathop {\longrightarrow }\limits ^{\gamma _{A}}\Phi , \hbox {B}\mathop {\longrightarrow }\limits ^{\gamma _{B}}\Phi \end{aligned}$$

where $\eta$, $\eta _s$, and $\eta _p$ represent the rates of three different division types of stem cells, $\omega _{p/s}$ denote the transition rates of *S*/*P* and $\lambda _p$, $\lambda _A$, $\lambda _B$, $\mu _d$, $\mu _A$, and $\mu _B$ denote the rates of six different division types that progenitor cells can go through. In addition, $\gamma _A$ and $\gamma _B$ indicate at which rate *A* and *B* cells diminish from the population. Considering $n_S$, $n_P$, $n_A$, and $n_B$, as the average densities of cell types *S*, *P*, *A*, and *B* respectively ($n_{S, P, B, A}$ are cell numbers normalized by volume), the time evolution of $n_{S, P, B, A}$ is given by

5 $$\begin{aligned} {\left\{ \begin{array}{ll} \partial _t n_S = n_S \eta _S - n_S \eta _P+n_P w_S -n_S w_P, \qquad \quad \quad \quad \quad \, \,\,\,\\ \partial _t n_P = n_S\eta +2n_S\eta _P+n_S w_P-n_P w_S \qquad \qquad \quad \quad \,\,\, \\ \qquad \qquad \quad \quad -n_P(-\lambda _P+\mu _d+\mu _A+\mu _B), \qquad \qquad \quad \quad \\ \partial _t n_A = n_P(\lambda _A+\mu _d+2\mu _A)-n_A\gamma _A, \qquad \quad \quad \qquad \quad \, \\ \partial _t n_B = n_P(\lambda _B+\mu _d+2\mu _B)-n_B\gamma _B.\quad \quad \quad \qquad \qquad \,\,\, \end{array}\right. } \end{aligned}$$

It was previously proven that this system can reach its stable steady state on $(n_A^*,n_B^*,n_P^*,n^*)$, as long as the following conditions are satisfied^3^:

6 $$\begin{aligned} \eta _S'(n)<0, \;\; and \;\; \lambda _P < \mu _d+\mu _A+\mu _B, \end{aligned}$$

where *n* is the average total density of cells, computed as $n = n_S+n_P+n_A+n_B$, and $\eta _S = \eta _S(n)$. We set the parameters in the model in a way that these two essential conditions are satisfied.

### Stem cells’ internal mechanism

The following set of ODEs used in several projects^2,20,28^ to describe a two-element bi-stable regulatory network, here is employed as the stem cells’ internal mechanism:

7 $$\begin{aligned} {\left\{ \begin{array}{ll} \frac{dx_s}{dt}=\iota _{x_s} \frac{\beta _s^n}{\beta _s^n+y_s^n}-\gamma x_s \\ \\ \frac{dy_s}{dt}=\iota _{y_s} \frac{\beta _s^n}{\beta _s^n+x_s^n}-\gamma y_s \\ \end{array}\right. } \end{aligned}$$

It actually provides the regulatory mechanism which takes care of the part of the model dynamics in Eq. (1). The form of the above-mentioned ODEs is stand on some the fundamental assumptions of the model: first, $X_s$ and $Y_s$ are the relative amount of two cell determinants, and as it is deducible from the phrase that their values determine the cell’s final fate, second, the mutual repression effect of the determinants (in the form of Hill functions), and their degradation rate describe the dynamical behavior of SCs. Here, *n*, $\beta _s$, $\iota _{X_s}$, $\iota _{Y_s}$, and $\gamma$ are set as the Hill coefficient, the effective rate of determinants synthesis, inhibition rates of $X_s$ and $Y_s$, and the degradation rate, respectively.

In general, the parameters are tuned (by try and error method) to build the SCs’ regulatory switch with two stable steady states corresponding to two main cell fates in the model, stem cell type (*S*) and progenitor cell type (*P*). In addition, to have the first condition in Eq. (6) be satisfied, as $\iota _{x_s}$ is the parameters which directly controls the rate of symmetric division of $\hbox {S}\mathop {\longrightarrow }^{\eta _{s}} \hbox {S} + \hbox {S}$, we need to set $\iota _{x_s}$ as a function of n, where $\iota _{x_s}(n)'<0$^3^.

### Progenitor cells’ internal mechanism

The regulatory mechanism of the progenitor cells is described by the following set of ordinary differential equations (ODEs) as a two-element tristable system^2,20,28^:

8 $$\begin{aligned} {\left\{ \begin{array}{ll} \frac{dx_p}{dt}=(\alpha _{x_p}+\varepsilon _{s_1})\frac{x_p^n}{\beta _p^n+x_p^n}+\iota _{x_p} \frac{\beta _p^n}{\beta _p^n+y_p^n}-\gamma x_p \\ \\ \frac{dy_p}{dt}=(\alpha _{y_p}+\varepsilon _{s_2})\frac{y_p^n}{\beta _p^n+y_p^n}+\iota _{y_p} \frac{\beta _p^n}{\beta _p^n+x_p^n}-\gamma y_p. \\ \end{array}\right. } \end{aligned}$$

Likewise, it is assumed that $X_p$ and $Y_p$ are two cytoplasmic determinants whose values determine the final fate of progenitor cells’ offspring^3^. Here in this system, *n*, $\alpha _{x_p/y_p}$, $\beta _p$, $\iota _{X_s/Y_s}$, and $\gamma$ are studied as the Hill coefficient, activation rates, the effective rate of determinants synthesis, inhibition, and degradation rate, respectively. There are two more parameters, $\varepsilon _{s_1}$, and $\varepsilon _{s_2}$ as “signaling effect coefficients” that will be discussed in this section. The parameters are set to build a tristable steady-state system with three fixed points corresponding to three cell fates, one progenitor (*P*), and two differentiated cell types (*A* and *B*). The phase plane of this tristable steady-state system represents the domains of the three cell fates. These domains indicate the decision boundaries governing the daughter cells’ final fate right after their birth^3^.

As discussed before, in the first and second phases of the simulation, a “leading” signal, coined as $S_l$, is produced to organize differentiated cells in order to form the desired pattern in the population. Besides, to maintain the population pattern in steady states, it is essential for each differentiated cell, *A*/*B*, to secrete their distinct signaling molecules, $S_1$, $S_2$ to conquer their territory^3^. As shown in Eq. (8), the impact of the signals $S_l$, $S_{1}$, and $S_{2}$ is performed on progenitor cells’ internal switch via $\alpha _{x_p}$, and $\alpha _{y_p}$ through $\varepsilon _{s_{1}} and \varepsilon _{s_{2}}$ parameters. The former’s impact arranges the population’s cells, while the latter maintains/fixes them in their territory.

The “signalling effect coefficient”, $\varepsilon _{s_i} = f(S_l)+g(S_i)$, where $i \in {1,2}$. The model primarily relies on $S_l$ to establish a spatial pattern in the population. This implies that it is unnecessary to retain it in the model once the pattern has been established. Therefore, here, $f(S_l) = \frac{a\;\;S_l^{12}}{1+S_l^{12}}$, in the absence of $S_{i}$, and $f(S_l) = 0$, otherwise. Furthermore, it is biologically reliable, since that is observed during development, and it minimizes the energy costs of the system. $g(S_i) = \frac{a S_i}{b}$, if $S_i\le b$, and $g(S_i) =a$, otherwise^3^. Finally, to have the second condition in Eq. (6) be satisfied, the parameter $\beta _p$ is tuned (by try and error method) in such a way that the rate of symmetric division, $\hbox {P}\mathop {\longrightarrow }\limits ^{\lambda _{P}} \hbox {P} + \hbox {P}$ is much less than divisions in the forms of $\hbox {P}\mathop {\longrightarrow }\limits ^{\mu _{d}} \hbox {A} + \hbox {B}$, $\hbox {P}\mathop {\longrightarrow }\limits ^{\mu _{A}} \hbox {A} + \hbox {A}$, and $\hbox {P}\mathop {\longrightarrow }\limits ^{\mu _{B}} \hbox {B} + \hbox {B}$ (see [Khorasani, and Sadeghi 2022]^3^ for details).

### Signalling dynamics

Here, the “leading” signal, $S_l$, has been studied in three ways. A fixed deterministic pattern is first considered. Second, the dynamic of the $S_l$ signal is described as follows:

9 $$\begin{aligned} \frac{ds_l}{dt}=D \nabla ^2s_l+\alpha _{s_l}-k s_l, \end{aligned}$$

where *D*, $\alpha _{s_l}$, and *k* are defined as diffusion, production and degradation rates. Third, to generate $S_l$ signal, a Turing system is used as follows^59^:

10 $$\begin{aligned} {\left\{ \begin{array}{ll} \frac{ds_l}{dt}=d_l\nabla ^2s_l+ \gamma _{s_l} f(s_l, s_a) \\ \frac{ds_a}{dt}=d_a \nabla ^2s_a+ \gamma _{s_l} g(s_l, s_a).\\ \end{array}\right. } \end{aligned}$$

**Figure 1:**
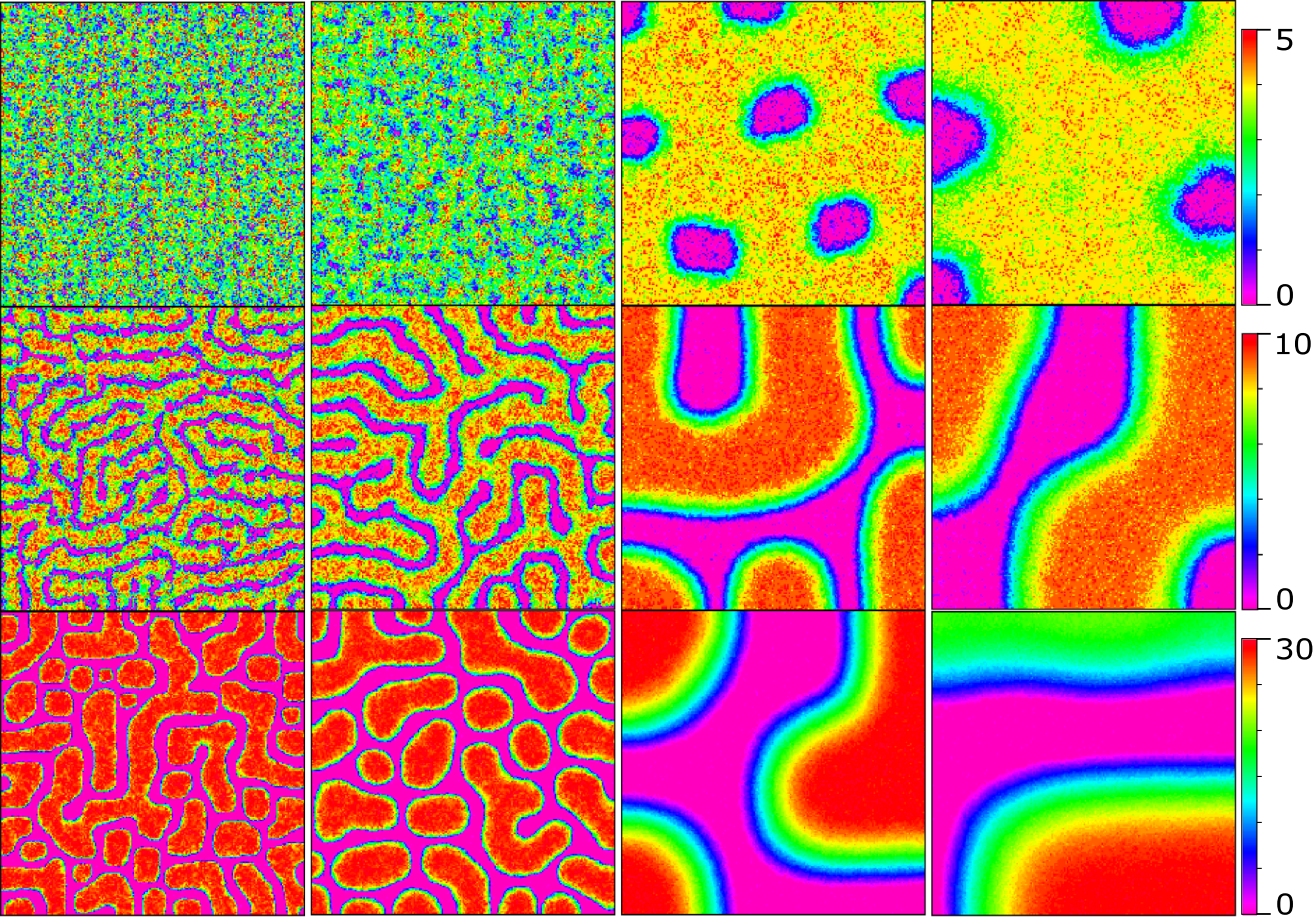
The Turing signal patterns of $S_l$, generated by Gillespie algorithm. From right to left, $(d_l, d_a) = (5e3, 1e5), (1e4,2e5),(2.5e5,5e6),(1e6,2e7)$, and from top to bottom, $s_{upper} = 5,10,30$, and $s_{lower} = 0$.

It is the general model of a Turing system that promises to generate spatially heterogeneous patterns with two diffusive chemicals ($s_l$ and $s_a$) from uniformly distributed sources. Here, $S_l$ demonstrates the number of the “leading” signal, and $S_a$ is the number of its associated substance which differs in diffusivity. The parameters, $d_{l/a}$, and $\gamma _{s_l}$ are diffusion coefficients and a common factor multiplied by the reaction terms. In Eq. (10), it is assumed that $f(s_l, s_a) = A\;s_l-s_a+C$, and $g(s_l, s_a) = B\;s_l-s_a-1$ as the linear reaction terms, where *A*, *B*, and *C* are constants. To observe the formation of stable patterns in a system with linear reaction terms, it is necessary to constrain the $S_l$ value within a finite range^59^. To be specific, it is assumed that $s_{lower} \le S_l \le s_{upper}$, where $s_{lower}$ $s_{upper}$ are constants. Since the presence of stochasticity is inevitable in our model, the Gillespie algorithm is used to create Turing patterns. Therefore, the model’s parameters had to be tuned (by try and error method), compared to the ones set in Shoji’s model^59^. Figure 1 shows the effect of parameters, $s_{upper}$, and $d_{u/a}$ on the Turing patterns generated by Gillespie’s algorithm. In Fig. 1, from right to left, $(d_l, d_a) = (5e3, 1e5), (1e4,2e5),(2.5e5,5e6),(1e6,2e7)$, and from top to bottom, $s_{upper} = 5,10,30$ ($s_{lower} = 0$). Patterns in the first, second, and third rows of Fig. 1 coined as reversed-spot, stripe, and spot patterns respectively^59^. A set of reaction-diffusion equations describe the dynamics of the signaling molecules, $S_1$, and $S_2$ as follows^3^:

11 $$\begin{aligned} {\left\{ \begin{array}{ll} \frac{ds_1}{dt}=D \nabla ^2s_1+\alpha _{s_1} \frac{\beta ^n}{\beta ^n+s_2^n}-k s_1 \\ \\ \frac{ds_2}{dt}=D \nabla ^2s_2+\alpha _{s_2} \frac{\beta ^n}{\beta ^n+s_1^n}-k s_2 \\ \end{array}\right. } \end{aligned}$$

Here *D*, *k*, $\alpha _{s_i}$ ($i \in {1, 2}$) represent the diffusion coefficient, the decay, and the production rate of the signaling molecules, respectively. Besides, *n* is the Hill coefficient, and $\beta$ is the effective rate of signaling molecules’ synthesis. The parameters are set to describe the dynamics of signaling molecules, $S_1$, and $S_2$, in the form of a bi-stable system^3^. In this model, for generating reversed-spot, stripe and spot signalling patterns, $(d_l, d_a)$ is set equal to (2.5*e*5, 5*e*6), (1*e*4, 2*e*5), (1*e*4, 2*e*5) and $s_{upper} = 5,10,30$ ($s_{lower} = 0$), respectively.

Here it is critical to address one fundamental question: How can *A* (*B*) cells maintain their territory? Promptly after the birth of *A* (*B*) cells in the population, the secreting of *S*1 (*S*2) signaling molecules is triggered. They defend the *A* (*B*) territory by first, preventing the production of *S*2 (*S*1) (based on the second term in Eq. (11)), and second, by increasing the birth rate of *A* (*B*) cells through the $\varepsilon _{s_1}$ ($\varepsilon _{s_2}$) in Eq. (8).

### Gillespie algorithm

The Gillespie algorithm is used to capture the time evolution of the system^2,3,9,29,60^. Table 1 illustrates 28 potential reactions that can occur in each time step, as well as their corresponding propensity functions and parameters’ values. As the introduced propensity functions represent high-order reactions, they are used as an approximation to the Gillespie algorithm^61^. The endpoint of the simulation is when the stem cells have gone through 50 divisions on average.

**Table 1:** The potential reactions of the system together with their corresponding propensity functions.

no	Reactions	Propensity Func.	no	Reactions	Propensity Func.
1	Production of $X_s$	$\iota _{x_s} \frac{\beta _s^n}{\beta _s^n+y_s^n}$ $\iota _{x_s} = 85, \beta _s = 45, n = 4$	15, 16	*A*/*B* cell death	$\gamma _{A/B}$ $\gamma _{A}= 0.003, \gamma _{B}=0.0034$
2	Production of $Y_s$	$\iota _{y_s} \frac{\beta _s^n}{\beta _s^n+x_s^n}$ $\iota _{y_s}=100,\beta _s = 45,n=4$	17	Production of $S_{1}$	$\alpha _{s_{1}} \frac{\beta ^n}{\beta ^n+s_{2}^n}$ $\alpha _{s_{1}}=220,\beta =2,n=4$
3, 4	Degradation of $X_s$/$Y_s$	$\gamma x$; $x \in \{x_s, y_s\}$ $\gamma =1$	18	Production of $S_{2}$	$\alpha _{s_{2}} \frac{\beta ^n}{\beta ^n+s_{1}^n}$ $\alpha _{s_{2}}=220,\beta =2,n=4$
5	SC. Division	$r_s$ $r_s=56.4$	19, 20	Degradation of $S_{1/2}$	$k s_{i}$; $i \in \{1,2\}$ $k=0.5$
6	SC. Transformation	$w_p$ $w_p=0.329$	21, 22	Diffusion of of $S_{1/2}$	$\frac{D}{h^2}$ $D=110,h=1$
7	SC. Movement	$m_s$ $m_s=2.82$	23	Production of $S_{l}$ in 2nd, and 3rd Scenario	$\alpha _{S_l}$, and $\gamma _{S_l}(AS_l+C$) $\alpha _{S_l} = 1e4, A =.9, C =.2$
8	Production of $X_p$	$(\alpha _{x_p}+\varepsilon _{s_1})\frac{x_p^n}{\beta _p^n+x_p^n}+\iota _{x_p} \frac{\beta _p^n}{\beta _p^n+y_p^n}$ $\alpha _{x_p}=30,\iota _{x_p}=30,\beta _p=47.5,n=4$	24	Degradation of $S_{l}$ in 2nd, and 3rd Scenario	$kS_l$, and $\gamma _{S_l} s_a$ $k = 0, \gamma _{S_l} = 1e4$
9	Production of $Y_p$	$(\alpha _{y_p}+\varepsilon _{s_2})\frac{y_p^n}{\beta _p^n+y_p^n}+\iota _{y_p} \frac{\beta _p^n}{\beta _p^n+x_p^n}$ $\alpha _{y_p}=30,\iota _{y_p}=30,\beta _p=47.5,n=4$	25	Diffusion of $S_{l}$ in 2nd, and 3rd Scenario	$D_{S_l}$, and $d_l$ $D_{S_l}=2.5e5$
10, 11	Degradation of $X_p$/$Y_p$	$\gamma x$; $x \in \{x_p, y_p\}$ $\gamma =0.38$	26	Production of $S_{a}$	$\gamma _{s_a}BS_l$ $\gamma _{s_a}=1e4,B = 1.2$
12	Prg. Cell Division	$r_p$ $r_p = 32.9$	27	Degradation of $S_{a}$	$\gamma _{s_a}(s_a+1)$
13	Prg. Cell Transformation	$w_s$ $w_s=0.1645$	28	Diffusion of $S_{a}$	$d_a$
14	Prg. Cell Movement	$m_p$ $m_p=0.94$			

The simulation starts with an initial population of stem cells distributed randomly in a hypothetical dish. Each individual mesh of the dish can be potentially occupied with one of the four cell types, *S*, *P*, *A*, or *B* with or without the presence of signals $S_l$, $S_1$ or $S_2$. The number of determinants is chosen randomly from the stem cells’ territory in the phase plane describing its dymanics^3^. In the first phase of the simulation, a mesh occupied with a stem cell can be the scene for eight (11 in the third scenario) potential reactions: production/degradation of determinants $X_S$, and $Y_S$, production/degradation/diffusion of $S_l$ signal (plus those of $S_a$ signal in the third scenario), and stem cells’ movement. As shown in rows 1 to 4 in Table 1, the propensity function of production/degradation reaction of determinant $X_S$ ($Y_S$) is determined based on the positive/negative term in the first (second) ODE in Eq. (7). The movement is provided in the model to prevent the system from being blocked (see Khorasani, and Sadeghi^3^ for details). As it is depicted in rows 7, and 14, the movement can occur at a constant rate ($m_s$, and $m_p$ for stem cells and progenitor cells, respectively), and this involves randomly selecting an empty mesh, and then placing the cell from the current mesh into that empty space. Rows 23 to 25 illustrate the propensity functions of production/degradation/diffusion of $S_l$ signal in the second and third scenario captured by Eqs. (9), and (10), respectively. In the same manner, rows 26 to 28 show the propensity functions of production/degradation/diffusion of $S_a$ signal in the third scenario. At each time step of the Gillespie algorithm, one of the above-mentioned reactions is chosen and based on that in the current mesh, one of the determinants $X_S$, and $Y_S$, or signal $S_l$ ($S_a$ in the third scenario) decreases/increases in value or in one of the neighboring meshes, the value of signal $S_l$ ($S_a$ in the third scenario) decreases/increases. It is necessary to mention that all these reactions also occur in the second and third phases of the simulation. However, they are the only ones with a chance to happen in the first phase.

The second phase of the simulation starts when the $S_l$ signal is organized in the desired pattern. In this model, it is triggered after a certain number of iterations from the start point. As we move into the second phase, two reactions with prominent roles are revealed: the division of SCs and the transformation of the SCs with constant rates of $r_s$, and $w_p$, respectively. With these two reactions, progenitor cells and as a result, specialized cells (*A* and *B* cells) and their corresponding signal molecules emerge in the population. In other words, all the reactions listed in Table 1 can play a role in the system’s scene from now on.

When a stem cell divides into a progenitor cell, the number of determinants $X_P$ and $Y_P$ in a new-born cell is initiated randomly from the middle domain of it’s corresponding phase plane^3^. The propensity functions of production/degradation reaction of these two determinants are captured based on Eq. 8 (see rows 8 to 11)^3^. A progenitor cell can be divided with a constant rate of $r_p$ (row 12) to *P*, *A*, and *B* cells. With *A* and *B* cells in the population, $S_1$ and $S_2$ signaling molecules can set foot in the system, and in a mesh occupied with $S_1$, and $S_2$, five reactions, production/degradation of $S_1$, and $S_2$, and diffusion got the chance to happen. Rows 17 to 22 demonstrate their corresponding propensity functions based on Eq. (11) (see [Khorasani, and Sadeghi 2022]^3^ for details).

For the rest of the reactions, the propensity functions are chosen as constant rates^3^. In the model, death is defined as the omission of the chosen cell from the population. During the division, two offsprings are located in the current mesh and one of the empty neighboring meshes. The number of determinants $X_1$, and $X_2$ ($Y_1$, and $Y_2$) in two new-born cells are set as $X_1 \sim B(\#X, 1/2)$, and $X_2 = \#X - X_1$ ($Y_1 \sim B(\#Y,1/2)$, $Y_2 = \#Y-Y_1$), where $\#X$ ($\#Y$) is the number of determinant in the mother cell^3^. The transition from a stem cell to a progenitor cell occurs with the constant rates of $w_p$, where during that the *S* cell in the current mesh is replaced with a *P* cell. The number of determinants $X_P$ and $Y_P$ in transformed cell is initiated randomly from its specific domain of it’s corresponding phase plane^3^. The transition from a *P* cell to a *S* cell occurs with the constant rates of $w_s$ with the same manner.

### Scoring algorithm

To evaluate the maintenance of the spatial patterns in the population, we used the “scoring algorithm”^3^. To implement this algorithm, we need to construct two filter matrices corresponding to the “leading” signaling pattern, namely template and penalty. For each initial “leading” signaling matrix, if a mesh value is greater than 250/2 (250 is the maximum value of leading signaling molecules in each mesh) the corresponding mesh’s values of the template matrix are equal to 1, and it is equal to $-1$, otherwise. The values of the outer-dish meshes are set to 0. The values of the penalty matrix are equal to 1 in out-of-dish meshes and are equal to 0 otherwise. Each simulation starts with an initial state with a desired spatial pattern and continues until all progenitor cells have undergone an average of 50(100) divisions. Out of all the states encountered during the simulation, we select 500(1000) snapshots with dimensions of $d \times d$. For each snapshot of the system state a corresponding $2d\times 2d$-dimensional matrix is created as follows: In each snapshot, meshes corresponding to yellow/red pixels (*A*/*B* cells) are set to $1/-1$, and the rest of the elements are set equal to 0. This $d \times d$ matrix makes the middle part of the corresponding $2d\times 2d$-dimensional matrix. The rest of the values are set equal to 0.

To measure the score values for each snapshot, we slide the template, and penalty filters over each of the 500(1000) $2d\times 2d$-dimensional matrices, from top-left to bottom-right (mesh by mesh), multiply their corresponding values one by one and compute the summations. Each time the filters are moved over the snapshot matrix (one mesh at a time), two values are computed-one for the template filter and another for the penalty filter. The subtraction of these values is calculated, and the resulting score assigned to each snapshot is determined as the maximum among all subtracted values obtained by filters’ sliding over the snapshot’s matrix. Finally, we normalize the score values in the range of [0,1] by dividing to the maximum score value (where the system state perfectly matches the template and penalty matrices).

## Results

### No “leading” signal

**Figure 2:**
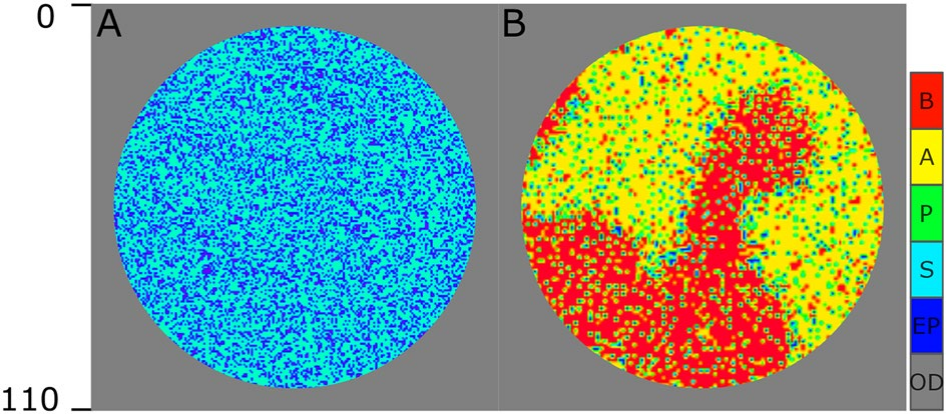
The initial and final state of the system in the absence of “leading” signal. Each pixel indicates one individual cell, S in cyan, P in green, (**A**) in yellow, and (**B**) in red, an empty position (EP) in blue, or an out-of-dish space (OD) in gray.

The computational model of SCs’ mechanism designed by Khorasani et al.^3^ can maintain the initial spatial pattern in the population. What if there is no initial pattern, and the initial state of the system is a population of randomly distributed stem cells? Two potential scenarios could happen: first, the four types of cells, *S*, *P*, *A*, and *B*, are randomly distributed in the population, second, in some parts of the dish, *P* cells divide into more *A*/*B* cells, certainly by chance, and then they maintain their territory by secreting $S_1$/$S_2$ signaling molecules to the end of system time. To address this question, the first simulation starts with a population of stem cells in the dish as shown in Fig. 2A, in which the *S* cells are shown in cyan and the blue and gray pixels represents the empty and out of dish positions as indicated in the colorbar. By implementing Gilespie algorithm and the reactions described in Table 1, we let the system be updated to the point that all the *P* cells have been through at least 50 divisions. Figure 2B illustrates the final state of the system containing all four cell types *S*, *P*, *A*, and *B* in cyan, green, yellow and red, respectively. The results support the second scenario as we expected. The specialized cells (*A*, and *B*) are randomly born in an area and defend that as their territory. It is worth-mentioning here that the initial state of all simulations are represented the same as the population of *S* cells randomly distributed in the dish.

### Fixed “leading” signal

**Figure 3:**
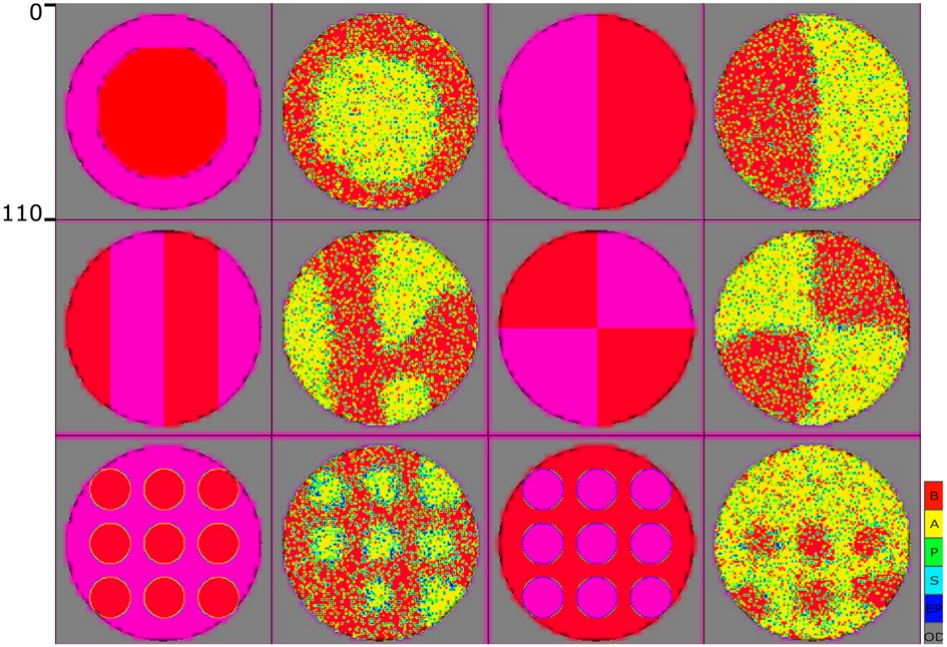
The presentation of static “leading” signal ($S_l$) (first, and third columns) and their corresponding patterns formation (second, and fourth columns) in the population. In the leading signal patterns, “Red” represents the value of 250, while “Magenta” represents the 0 value. In the second and fourth columns, as it is shown in the colorbar, each pixel indicates one individual cell, one individual cell, S in cyan, P in green, A in yellow, and B in red, an empty position (EP) in blue, or an out-of-dish space (OD) in gray.

At the beginning of the development process, in the presence of BMP4, hESCs are manipulated to produce an ordered array of specialized cells. Finally, they generate spatial patterns in the population^30^. It is evidently concluded that the presence of a “leading” signal, imitating BMP4’s role, is essential for the formation of patterns in the population. Here, the second experiment is designed to demonstrate that starting with a population of stem cells, our designed system has the potential to generate an ordered array of differentiated cells in response to an initial “leading” signal. In the second experiment, fixed patterns of $S_l$ signals are assumed as the “leading” signal as shown in the first and third columns of Fig. 3 in which the “Red” and “Magenta” colors represent the maximum and zero values of signalling molecule in the corresponding mesh. The results in the second and fourth columns show that the model can effectively self-organize in response to the “leading” signal. Spot and reversed-spot patterns could not be perfectly formed in the population. Although it is expected^3,31^, since the territory of one side is dominant^3,31^, which makes it easier for dominant cells to invade the apposite side.

### Dynamic “leading” signal

**Figure 4:**
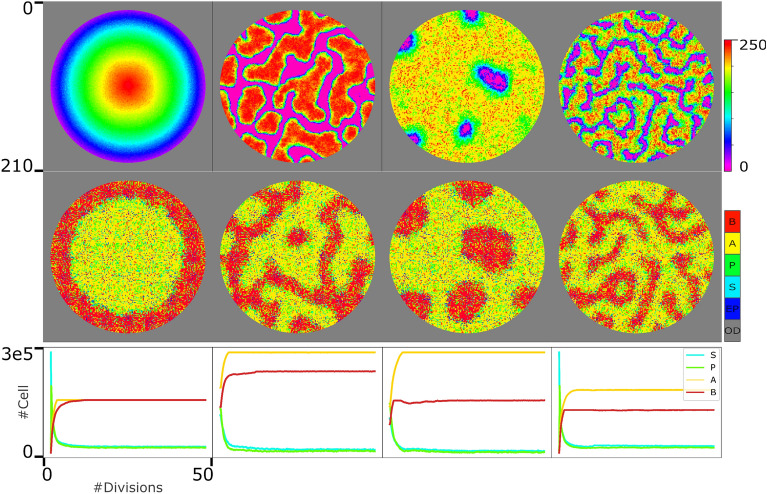
The system behavior in the face of dynamic “leading” signals.The first row represents the Gaussian, spot, stripe, and reversed-spot patterns as the “leading” signals, $S_l$. The values of the signaling molecules are scaled in the range of [0, 250] to be in agreement with the values of static leading signals. The second row demonstrates the system state after 50 divisions (of progenitor cells). Each pixel in the second row can potentially demonstrates *S*, *P*, *A*, *B* cell types, an empty position or an out-of-dish space as shown by the colorbar in the second row. The third row shows the abundance of four cell types in the population through the simulation.

In the third experiment, we challenge our model with dynamic, biologically reliable “leading” signals. Besides, in this experiment, the “leading” signal does not last to the end point of the simulation but for a limited duration to initiate pattern formation in the population (at the end of phase $\#2$). It is in agreement with biological experiments done previously to recapitulate spatial patterning in early development^30^. For this purpose, here we used four dynamic patterns, Gaussian, Spot, reversed-Spot, and Stripe, as the “leading” signal, shown in the first row of Fig. 4^59^ in which the colorbar indicates the number of signalling molecule is each grid.

To study the capability of the model in initiating and maintaining the desired spatial pattern in the population and in the presence of dynamic “leading” signaling molecules, the medium is populated with randomly distributed SCs. The dish radius is initially set to 100 in the sense that any cell would be restricted to a maximum distance of 100 pixels from the center point. To compute the time evolution of the cell populations, a stochastic simulation using the Gillespie algorithm is applied. Simulation is terminated when the progenitor cells have gone through 50 divisions on average. A total of four simulations were run corresponding to four different types of “S” signals, Gaussian, Spot, reversed-spot, and Stripe. In the first phase of each simulation, SCs do not divide. Though, they are involved in producing the required“leading” signal based on Eq. (9) for Gaussian pattern and Eq. (10) for Turing patterns. The first row in Fig. 4 demonstrates the state of the “leading” signal, $S_l$, at the end of phase $\#1$. Until the end of phase $\#1$, the state of SCs in the dish remains unchanged and the same as the dish state in Fig. 2A. In the second phase, it is different. By triggering division in the second phase of the simulation, progenitor cells, and subsequently differentiated cells, A and B, emerge.

A and B cells in our model are self-organized to generate the desired spatial pattern in the population. The internal mechanism of *P* cells is designed to achieve this (Eq. 8). Based on Eq. (8), in the parts of the dish with maximum/minimum value of “leading” $S_l$ signal, the number of $X_P$/$Y_P$ determinants exceeds the number of $Y_P$/$X_P$ ones in *P* cells, and as a result with a great probability, *A*/*B* cells are born in these domains. Therefore, a pattern corresponding to the initial $S_l$ signal is generated in the population. The state of the system at which all progenitor cells have been through at least 50 divisions is shown in the second row of Fig. 4. In all four simulations, specialized cells, *A*, and *B* properly self-organize to create the desired pattern. The subplots in the third row of Fig. 4 show the diagrams of the abundance of four types of cells from the beginning to the end of the simulation. All diagrams have saturated so fast, and it indicates that the system can easily reach its stable, steady state on $(n_A^*,n_B^*,n_P^*,n^*)$ as proved and promised in Section “Mathematical model of the system”.

### Death rate

Here, we study the death rate values of *A* and *B* cells ($\gamma _A$ and $\gamma _B$). In this model, we have tuned more than 40 parameters (by try and error method), where all of them except one ($\gamma _{A/B})$ orchestrate the production rates in the population. The balance between production and death rates determines the abundance of cells in the tissue. The parameter $\gamma _{A/B}$ is the only parameter to control cells’ removal from the system. Therefore, the $\gamma _{A/B}$ value is critical in determining the *n* value in our system.

On the other hand, it was previously proven that this system could reach its stable, steady state on $(n_A^*,n_B^*,n_P^*,n^*)$, as long as the conditions in Eq. (6) are satisfied^3^. The parameters in the model are set in a way that both of these essential conditions are satisfied. However, the satisfaction of these conditions is independent of the $\gamma _{A/B}$ value. In other words, different values of the $\gamma _{A/B}$ cannot affect the system’s stability. Therefore, theoretically any positive value for the $\gamma _{A/B}$ is acceptable. However, the $\gamma _{A/B}$ value directly impacts the abundance of different cell types in steady state, i.e., $n_A^*$, $n_B^*$, $n_P^*$, and $n^*$ values. As to form a desired pattern, a specific number of $n_A^*$, $n_B^*$, $n_P^*$, and $n^*$ is certainly needed, one could easily conclude that the formation of a desired pattern directly depends on the $\gamma _{A/B}$ value in our model (keeping in mind that the rest of the parameters, with a key role in the production rate, are fixed). On the other hand, based on the time evolution of $n_A$, and $n_B$ in Eq. (5), $\gamma _A = (n_P^*(\lambda _A+\mu _d+2\mu _A))/n_A$ and $\gamma _B = (n_P^*(\lambda _B+\mu _d+2\mu _B))/n_B$. It makes it clear that $\gamma _{A/B}$, and $n_P^*$ values are both mutually dependent. However, at the beginning of the simulation, we do not have access to the value of any of them to fix the other one. Even knowing one of these values, we cannot determine the exact value of the other one. To be specific, due to the stochastic nature of the system, it is impossible to obtain the exact values of $\lambda _{A/B}$, $\mu _d$, and 2$\mu _{A/B}$ through simulation.

Looking on the bright side, we can approximate the $n_A^*$ and $n_B^*$ values based on the initial pattern of $S_l$, the “leading” signal. Assume that in an initial pattern (the first row of Fig. 4) *p* percent ($(1-p)$ percent) of the total positions are red/yellow (green/cyan/blue/magenta), the expected territory of *A* (*B*) cells. By utilizing trial and error method, to get a correct pattern in the populating corresponding to the initial $S_l$ pattern, the number of stem cells and progenitor cells with empty positions can not exceed 15 percent of the total dish’s positions. It is instantly concluded that the desired pattern occurs in the presence of $p*0.85*(N)$ ($(1-p)*.0.85*(N)$) number of *A* (*B*) cells in homeostasis state. In other words, $n_A^*=p*0.85*(N)$ ($n_B^*=(1-p)*.0.85*(N)$), where *N* is the number of all pixels in the dish. With this approximated value of $n_A^*$, and $n_B^*$ we set the $\gamma _{A/B}$ value as follows:

12 $$\begin{aligned} \gamma _i = {\left\{ \begin{array}{ll} 0.05 \qquad if \quad n_i < n_i^* \\ 0.1 \qquad \qquad \quad \; o.w.\\ \end{array}\right. } \end{aligned}$$

where $i \in {A, \; B}$. It is worth mentioning here that the $\gamma _A$, $\gamma _B$ values reported in Table 1 are the average values computed through the whole simulation. The results shown in Fig. 4 indicate that with this method of determining $\gamma _{A/B}$ value, the formation of the desired pattern as well as reaching and maintaining the stable, steady state on $(n_A^*,n_B^*,n_P^*,n^*)$ are guaranteed in our model.

**Figure 5:**
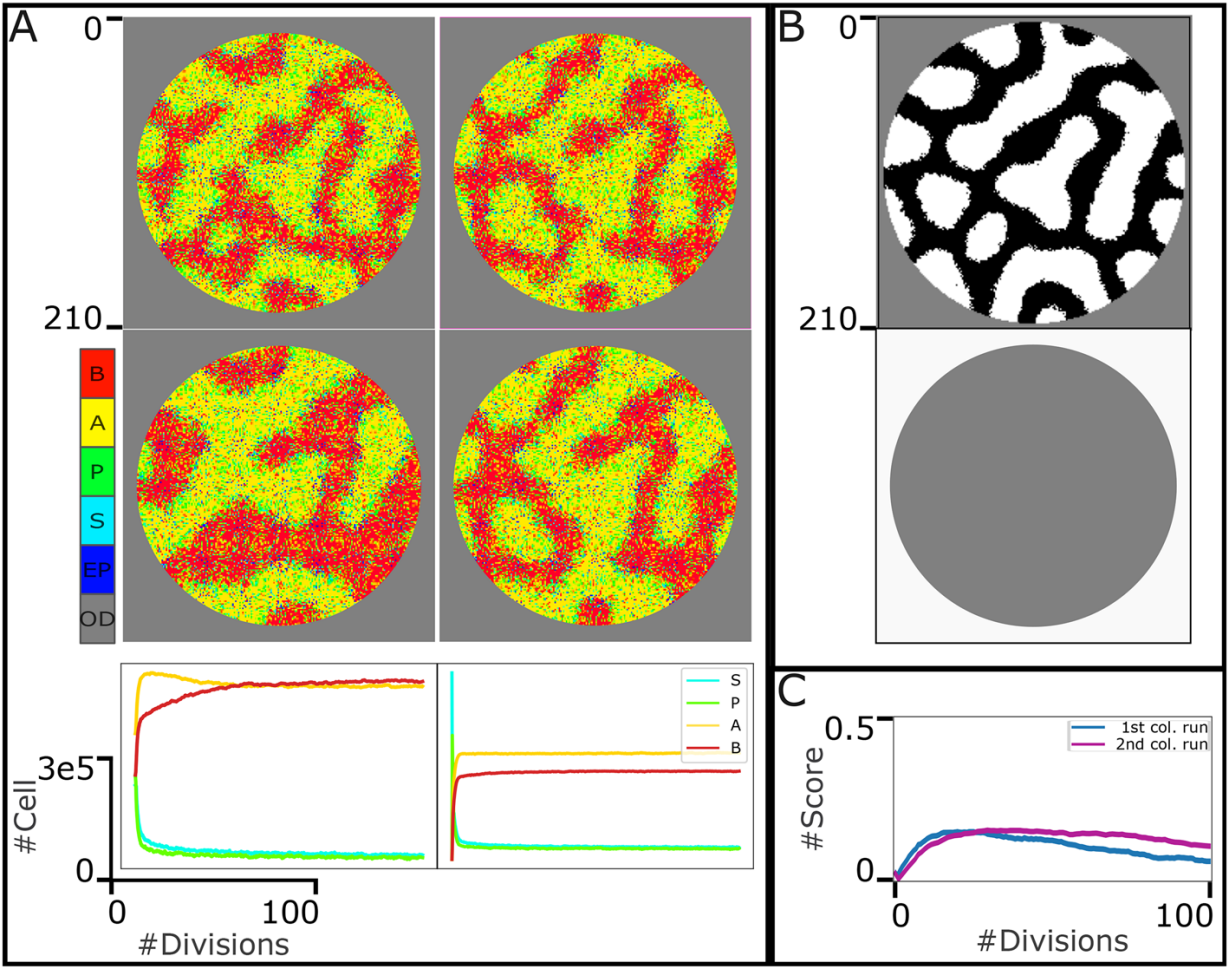
The system behaviour in the face of dynamic “leading” signals, $S_l$ with spot pattern. A. The first and second rows represent the system state after the 50 and 100 divisions (of progenitor cells), respectively. The first and second columns represent two different formulations of death rate: $\gamma _i = \frac{n_i}{n_i^*}*v_g$, and $\gamma _i = \root 3 \of {\frac{n_i}{n_i^*}}*v_g$, where $i \in {A, \; B}$. As it is shown in the colorbar, each pixel can indicate one individual cell, S in cyan, P in green, A in yellow, and B in red, an empty position (EP) in blue, or an out-of-dish space (OD) in gray. The third row shows the abundance of four cell types through the simulation. B. The template and penalty filters of spot signal pattern. C. The scoring traces corresponding to the first and second columns in panel A. It indicates how the pattern in population in different states of the system through time is aligned with the desired initial signalling pattern.

As the stability of the system was proved previously, one can instantly conclude that if we could calculate the average value of $\gamma _{A/B}$, accurately, based on Eq. (12), and substitute it for the death rate of *A*/*B* cells in the model, we would reach the same steady state of our system on $(n_A^*,n_B^*,n_P^*,n^*)$. However, the dynamics of the system would certainly differ. Aiming to find a suitable solution to determine $\gamma _{A/B}$ value, we studied two equations of $\gamma _A = n_P^*(\lambda _A+\mu _d+2\mu _A)/n_A$, and $\gamma _B = n_P(\lambda _B+\mu _d+2\mu _B)/n_B$. According to these equations, $\gamma _{A/B}$ can be determined based on the ratio of $n_{A/B}$ (calculated by multiplication of $n_P$, and production rate of *A* cells in the population), to $n_A^*$. To imitate the behavior of this dynamic, in the fourth simulation, we set the $\gamma _i = \frac{n_i}{n_i^*}*v_g$, where $i \in {A, \; B}$, and $v_g = 0.0125$ (Running different simulations, we reached the $v_g$ value. Using this $v_g$ value, the system reaches almost 15 percent of total dish pixels occupied by stem cells, progenitor cells, and empty positions. The rest of the fourth experiment is designed and run similarly to the third one. Figure 5A, the first column from top to bottom, shows the states of the system after (almost) 30, and 100 divisions, and the abundance of four cell types (through time) respectively. Here, the “leading” signal is spot pattern and the $\gamma _{A/B}$ value is set based on the ratio of $n_{A/B}$, and $n_{A/B}^*$.

The result shows that although the desired pattern is formed, it is not maintained properly in the system. The narrow regions of each domain (*A*/*B* domains) are merged. The reason is that in this last method of determining $\gamma _{A/B}$ value, there is a meaningful delay between the detection of a change in the number of cells, and the corresponding regulation of $\gamma _{A/B}$ value. In other words, a great difference between $n_{A/B}$, and $n_{A/B}^*$ is needed to regulate the $\gamma _{A/B}$ value. Therefore, there is always this chance that the cells on one side grow in number and capture the other side’s domain before the cells on the other side get the chance to defend their territory. For this reason, we modified the $\gamma _{A/B}$ equations to increase the sensitivity of the system: $\gamma _i = \root 3 \of {\frac{n_i}{n_i^*}}*v_g$, where $i \in {A, \; B}$. We repeated the experiment with this new formulation of $\gamma _{A/B}$ value for spot pattern as the “leading” signal in the population. The results are shown in the second column of Fig. 5A where the first, and second subplots show the state of the system after (almost) 30, and 100 divisions and the third subplot demonstrates the abundance of four cell types through the simulation. The results illustrate that this modification affects the formation of more proper Spot patterns in the population compared to the former one (first column in Fig. 5A). It is also worth mentioning here that the traces of the cell types’ abundance (third row in Fig. 5A) are saturated and it confirms the stability in the model which was promised in Section “Mathematical model of the system”.

### Maintenance of the Spatial Pattern in the population in addition to the cells’ abundance

To evaluate our model’s capacity for pattern maintenance by numbers, we leveraged the scoring method introduced by Khorasani et al.^3^. Figure 5B shows the template and penalty matrices for the spot pattern. For both experiments in Fig. 5A, during the simulation, we captured 500 snapshots of the model’s state. Then for all the selected snapshots, we slide the template and penalty matrices on the snapshot matrices and the corresponding scores (discussed in section 1.6) are calculated. Fig. 5C indicates the scoring traces of the first and second simulations in Fig. 5A in blue and magenta. In other words, the scoring algorithm compares each snapshot with the filter chosen corresponding to the final state of the $S_l$ signal in phase $\#1$, and the scoring traces demonstrated in Fig. 5C indicate how much the structural pattern in the population differs from the “leading” signal’s pattern through the simulation. Comparing two traces in this subplot also indicates that with the second formulation of gamma, the spatial pattern is maintained more properly in the population.

**Figure 6:**
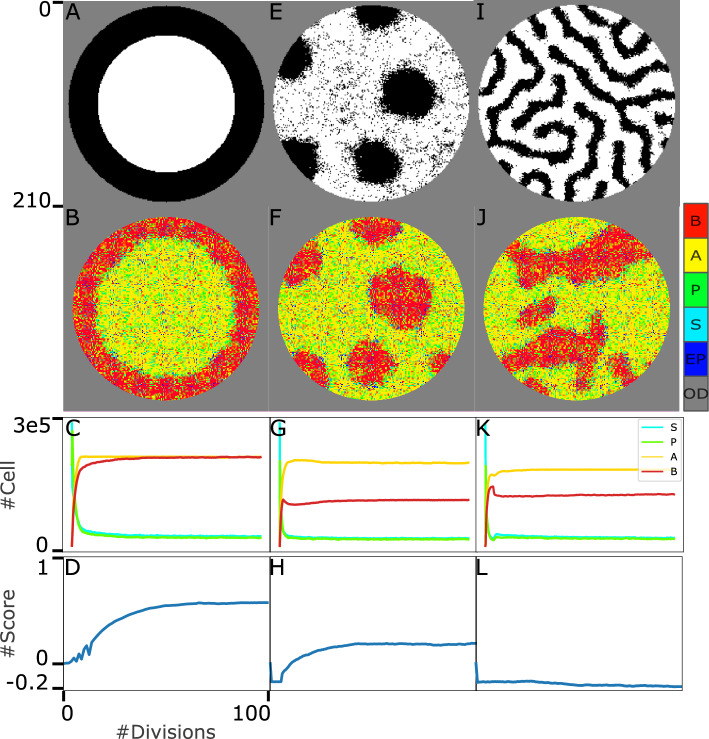
The system behaviour in the face of dynamic “leading” signals, $S_l$ with Gaussian, reversed-spot and stripe patterns. The first and second rows represent the template matrices and the system state after 100 divisions (of progenitor cells), respectively. As it is shown in the colorbar, each pixel can indicate one individual cell, S in cyan, P in green, A in yellow, and B in red, an empty position (EP) in blue, or an out-of-dish space (OD) in gray. Here, $\gamma _i = \root 3 \of {\frac{n_i}{n_i^*}}*v_g$, where $i \in {A, \; B}$. The third row shows the abundance of four cell types through the simulation. The fourth row demonstrates the scoring traces to evaluate the pattern maintenance in the population. It indicates how the pattern in population in different states of the system through time is aligned with the desired initial signalling pattern.

To evaluate the maintenance of the Spatial Pattern in the population with Gaussian, reversed spot and stripe signaling patterns, three more sets of simulations was run. In Fig. 6, from top to bottom, the template matrices (for the scoring algorithm), the final state of the system after almost 100 divisions, the abundance of four cell types, and the score traces through the simulation are shown. For reversed-spot and Gaussian patterns, the scoring traces are saturated (Fig. 6D,H). It can be interpreted as the proper maintenance of the established pattern in the population. The interpretation is also confirmed by the results shown in the Fig. 6B,F. The scoring trace for stripe patterns decreases rapidly after pattern formation (Fig. 6L). This behavior matches the system’s state shown in the Fig. 6J. In other words, in the face of the stripe pattern, pattern formation could occur however, it cannot be maintained in the population. As shown in Fig. 5C, the system behavior in the case of spot pattern differs from three other cases (after pattern formation). The spot pattern scoring diagram is not saturated like reversed-spot and Gaussian patterns, and it also does not decline rapidly like stripe patterns. It decreases so slightly. So slightly in the sense that even after 100 divisions, the loss of pattern in the population cannot be visually detected (Fig. 5A, second column). The scoring traces for the second and third experiments are also calculated and show in Supplementary Figures S1, and S2.

### Self-organization in the absence of fixed border

Our goal in this section is to test the model’s capabilities by studying system behaviour without a dish wall as a model’s constraint. Dish walls act like a template in the simulations. Biological cells, however, do not require templates to create structures. More precisely, in this experiment, the cells are not restricted within the boundaries of the dish. In this simulation, it is assumed that the physical boundary of the medium is infinite. In a part of the medium, stem cells are planted in a circle, then the first phase of the simulation begins.

To perform this simulation, we equipped the model with a pseudo-adhesion factor. In biological experiments, adhesion is a factor to ensure both cell and cell, and cell, and tissue cohesion (through cell-cell adhesion, and cell-matrix adhesion). Cadherins are trans-membrane proteins that mediate cell-cell adhesion. Multi-protein structures mediate cell-matrix adhesion. These protein-based macromolecules are mainly produced locally by cells. With mediating attachment they can control proliferation, division, apoptosis, and travel, and through these processes facilitate tissue homeostasis.

For simplicity, we do not consider an additional factor as a multi-protein structure responsible for adhesion in the model. Rather, we assume that the signaling molecules produced in the model also plays the role of these proteins. The simulation is mainly similar to what was discussed in Section “Dynamic “leading” signal” with some minor changes:

- Cells can move and be born outside the hypothetical borders of the initial population,
- It is assumed that initial cells in the population make an extracellular matrix,
- The cell-matrix adhesion is mediated by signaling molecules,
- With no cell-matrix adhesion division is inhibited, apoptosis is triggered, and migration is restricted (cells cannot easily move outside the matrix).

**Figure 7:**
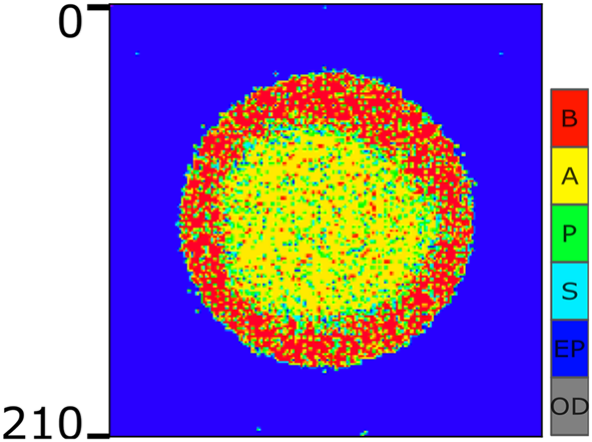
The system behabior in the face of Gaussian “leading” signal pattern, and in the absence of physical dish borders. As it is shown in the colorbar, each pixel can indicate one individual cell, S in cyan, P in green, A in yellow, and B in red, an empty position (EP) in blue, or an out-of-dish space (OD) in gray.

The result is shown in Fig. 7. Here, the “leading” signal, $S_l$, is considered in a Gaussiann pattern. Although the outer border is crooked and a limited number of cells are observed outside the borders, the initial desired pattern in the population is clearly observed. These results show the capability of the introduced model in formation of the desired pattern, even in the absence of a template.

## Discussion

Here, in this project the overall behaviour of the population is orchestrated by the internal decision-maker of stem cells, in the presence of stochasticity at the entire levels of the system, and without using any template or considering any movement process. An organism’s life depends on the precise functionality of its organs. Besides, a proportionally organized structure is essential to the organs’ proper functioning^3^. There is still much to be done to develop a comprehensive model that controls a stem cell’s decisions in starting from one cell, and ultimately generating and maintaining a proper structure in the final developed tissue. In our last project, we took a step toward this tempting big picture and introduced a simple model that could properly maintain the pattern in the tissue. Here, we took another step to imitate the early development processes of production and rearrangement. Through these two main processes a mass of homogeneous cells gives rise to a desired spatially ordered sequence^46^ as the final state of the system. In this model the initial mass of homogeneous cells is presented as a population of stem cells (*S*).

The model is described in Eqs. (1, 2, 3, 4), in details, and is simulated in four phases. The computation in the rest of the section 1.1 proves that in the second phase of the simulation, the described system could reach, and maintain the homeostatic state on $(n_A^*, n_B^*, n_P^*, n^*)$ under some reachable conditions. Besides, Eq. (5) indicates that theses four values are determined by the set of parameters in the model. By beginning with a population containing source cells, and choosing the right set of parameters, it proves that any desired spatial pattern can be organized using the right ratio of stem cells, specialized cells, and progenitor cells ($n_A^*, n_B^*, n_S^*, n_P^*$). However, achieving this desired pattern in the population requires a corresponding signal pattern to break the symmetry in the population. The Turing patterns were used as “leading” signals for this purpose. We also equipped the model with a factor to receive this “leading” signal and translate it into the intended pattern. The results reflect the capacity of our model to describe the behaviour of hESCs in pattern formation during early development.

In this model, two sets of ordinary differential equations are defined to describe the internal regulatory mechanism of stem cells (Eq. 7), and progenitor cells (Eq. 8)^3^. Previous studies^3^ reported that regulatory networks can provide a balance between proliferation and differentiation, as well as heterogeneity in populations. However, pattern formation could only happen in response to a “leading” signal^30^ to break the symmetry in the initial cell mass, and direct the population toward the desired pattern. The results support this fact. The results indicate that first, with no “leading” signal a random pattern is formed in the population (Fig. 2), and second, the formation of any desired pattern in the population is possible in the presence of its corresponding “leading” signal (Fig. 3). Although the “leading” signals in Fig. 3 prove the capability of the model in pattern formation, the signalling patterns are not biologically reliable. Studying the system behaviour in Fig. 4, indicates the capability of the designed model in pattern formation, as well as reaching and maintaining the stable steady state of the system on $(n_A^*, n_B^*, n_P^*, n^*)$ even in the face of stochastic, and dynamic “leading” signals.

In the scene of multi-cellular organisms, the production of cells is not the only key role in the development, and maintenance of the organizes tissues^46^. The process of apoptosis (programmed cell death) contributes greatly to this task by carefully balancing production and elimination in each tissue (with specific size, and shape). Multicellular organisms need apoptosis to reach homeostasis, and an excessive or insufficient apoptosis rate may cause dysfunctional tissues or tumorigenesis^46^. Therefore, in this project, we assigned a critical role to cell death rate as a control parameter. The first, and second rows of Fig. 5A show the corresponding states of the designed system (after 50, and 100 divisions, respectively) that approve this prominent role. Regarding the stability of the stripe pattern within our model, upon studying the results, it becomes evident that while our model is successful in pattern formation, it encounters challenges in maintaining the stripe pattern. This deficiency arises from the inherent vulnerability of narrow cell territories, where cells struggle to defend their domains, providing opportunities for rival cells to penetrate. Notably, due to the same diffusion rates of signaling molecules in the borders between the domains of cell types *A* and *B*, there is a facile interpenetration of territories, as visually depicted in the figures. In the context of the stripe pattern, wherein both cell types exhibit narrow territories, these territories essentially act as borders. Consequently, under certain conditions, *A* cells encroach upon the territory of *B* cells, and vice versa. This incursion disrupts the proper maintenance of patterns within the population.

In the third row of Figs. 5A, and 6 the abundance of all four cell types from the beginning to the end of the simulation is shown (and also in the last row of Fig. 4). In all cases, the cell types diagrams are saturated, which confirms the stability in the model which was proved and promised in the section 1.1.

The last row of Figs. 5A and 6 (as well as sumlementary Figures 1, and 2) shows scoring diagrams based on the borrowed algorithm introduced by Khorasani, et al. ^3^. The diagrams of Gaussian, and reversed-spot patterns are saturated (after pattern formation) which indicates the capacity of our designed model in maintenance of the organized pattern in the population (in addition to pattern formation, and reaching and maintenance of demanded cell abundance). However, the diagrams of both spot and stripe patterns decline over time. As discussed earlier in this section, the territory corresponding to these patterns is not wide enough to be maintained.

It is worth mentioning here that the magenta scoring trace of the spot pattern declines slightly in a way that it cannot be visually detected in the Fig. 5C. From the stability point of view, we need to facilitate the model to a different reaction-diffusion equation to describe the $S_1$, and $S_2$ behaviour in order to provide a great “defensive shield” for narrow territories (i.e. to provide saturated scoring diagrams). Biologically, this slight decline indicates that the pattern is not perfectly maintained but still satisfactory (in a spot pattern). Satisfactory in the sense that it maintains the pattern to the degree that the tissue can faithfully maintain its functionality. This interpretation is biologically reliable because the form of organs is constantly changing in living organisms. There is no organ in your body that remains completely similar. However, until some point it remains satifactorily the same and fulfills its functioning and not afterwards.

Lastly, a noteworthy point pertains to Fig. 7. Our model’s distinctiveness lies in its ability to coordinate the entire system’s behavior without relying on external control parameters. In our latest experiment, we examined the system’s behavior in the absence of a dish wall. In multicellular organisms, direct interactions and the extracellular matrix are pivotal in holding cells together, fostering cohesion. This cohesion, mediated by proteins secreted in the extracellular matrix, governs the formation of organized multicellular structures. In the absence of a dish wall, our model considers the simplest form of cohesion, as detailed in Section 2.6. The outcome illustrated in Fig. 7 highlights that even without the constraint of a dish border, we can effectively regulate the overall behavior of the system.

In order to ensure the proper functionality of a tissue, it’s fascinating to design a model that can form and maintain a well-organized structural pattern in the tissue. This will guarantee life. We are aware of the fact that here, we designed a simplified replica of two main processes of production, and rearrangement during early development. Pattern formation is much more complex and it occurs along a hierarchical path starting from one totipotent cell. Thus, in future work, it is essential to investigate stem cells’ strategies to reach a developed, organized structure along a hierarchical path. This strategy should provide a finite number of cycles with the following instructions: first, producing the demanded abundance of cells, second, secreting a signal third, breaking the existing symmetry in the population and fourth, producing the differentiated cells as the source of the next level of development in a spatially ordered sequence. It could be a promising step toward the big picture of this project: generating organoids starting from one stem cell.

### Supplementary Information

Supplementary Information.

## Data Availability

The software used to run all simulations was Python. The scripts and the data that support the findings of this study are available from the corresponding author upon request.
