# Supplementary material for: A computational model of stem cells’ internal mechanism to recapitulate spatial patterning and maintain the self-organized pattern in the homeostasis state
Source: Sci Rep. 2024 Jan 17;14:1528. doi: 10.1038/s41598-024-51386-z (PMC10794714; doi:10.1038/s41598-024-51386-z)
Supplement: Supplementary file 1 — Supplementary Information. [file 41598_2024_51386_MOESM1_ESM.zip › SupplementaryMaterial_NajmeKhorasani/SupplementaryMaterial_NajmeKhorasani.pdf]

A computational model of stem cells' internal mechanism to recapitulate spatial patterning and maintain the self-organized pattern in the homeostasis state

Najme Khorasani[1*] **and Mehdi Sadeghi**[2]

[1]School of Biological Sciences, Institute for Research in Fundamental Sciences (IPM), Tehran, Iran
[2]National Institute of Genetic Engineering and Biotechnology (NIGEB), Tehran, Iran
[*]najme.khorasani@ipm.ir

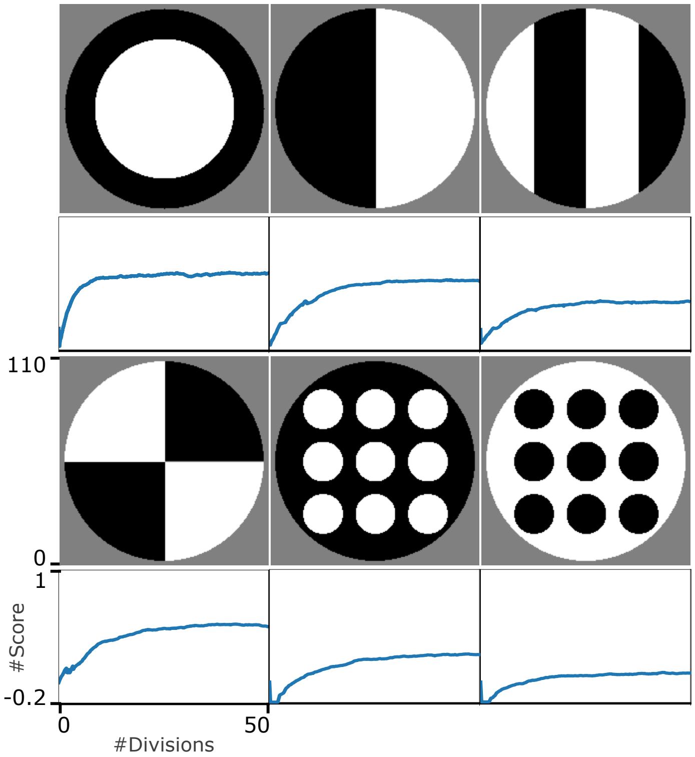

Figure 1. The scoring traces for the fixed leading signals in the second designed experiment. The template matrices for the scoring algorithm are shown in top rows of each paired subplots, where the scoring traces through 50 divisions are shown in bottom subplots.

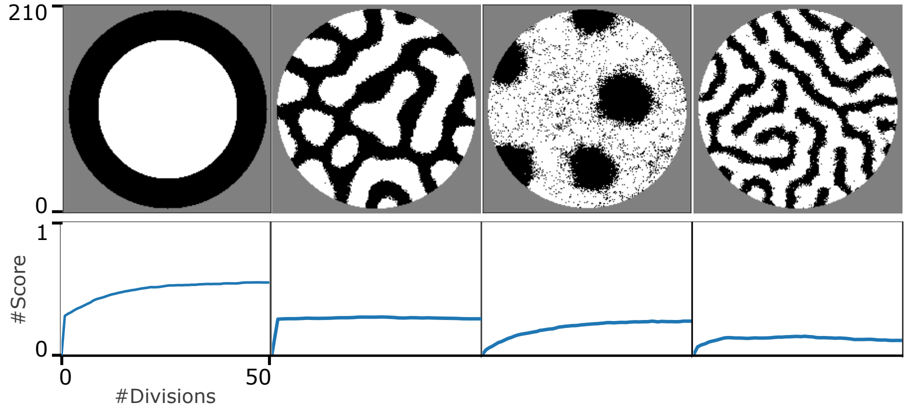

Figure 2. The scoring traces for the Gaussian, spot, reversed spot, and stripe patterns as leading signals, s_l, in the third designed experiment. The template matrices for the scoring algorithm are shown in top rows of each paired subplots, where the scoring traces through 50 divisions are shown in bottom subplots.
