# Supplementary figures and images for: A computational model of stem cells’ internal mechanism to recapitulate spatial patterning and maintain the self-organized pattern in the homeostasis state
Source: Sci Rep. 2024 Jan 17;14:1528. doi: 10.1038/s41598-024-51386-z (PMC10794714; doi:10.1038/s41598-024-51386-z)

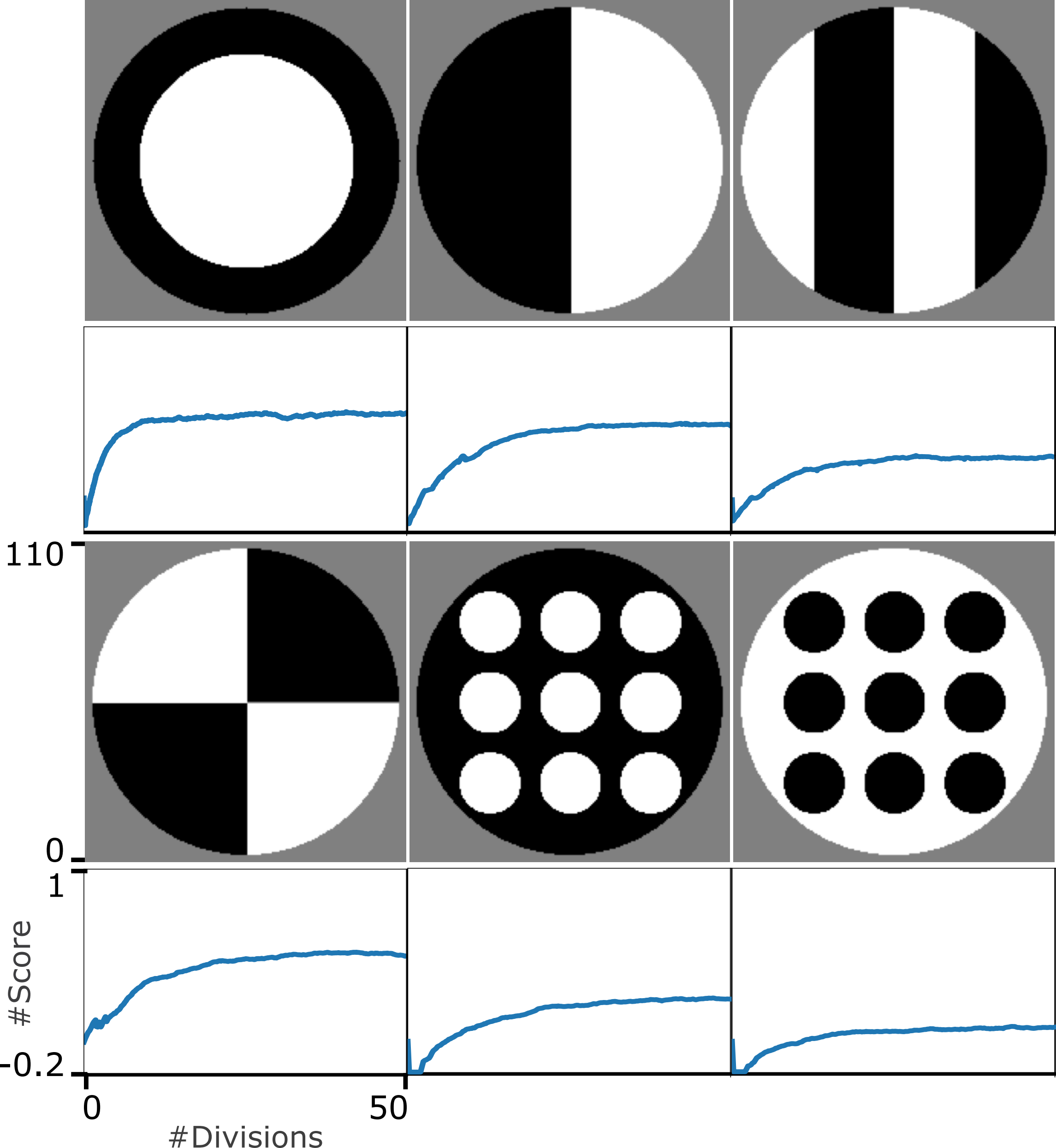

Supplement: Supplementary file 1 — Supplementary Information. [file 41598_2024_51386_MOESM1_ESM.zip › SupplementaryMaterial_NajmeKhorasani/2-img/Figure S1.pdf]

210

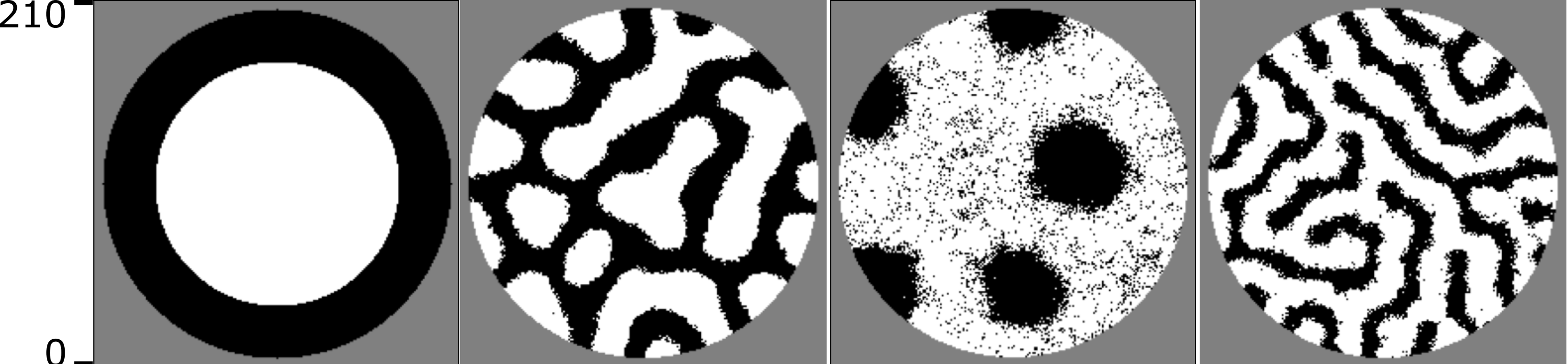

0

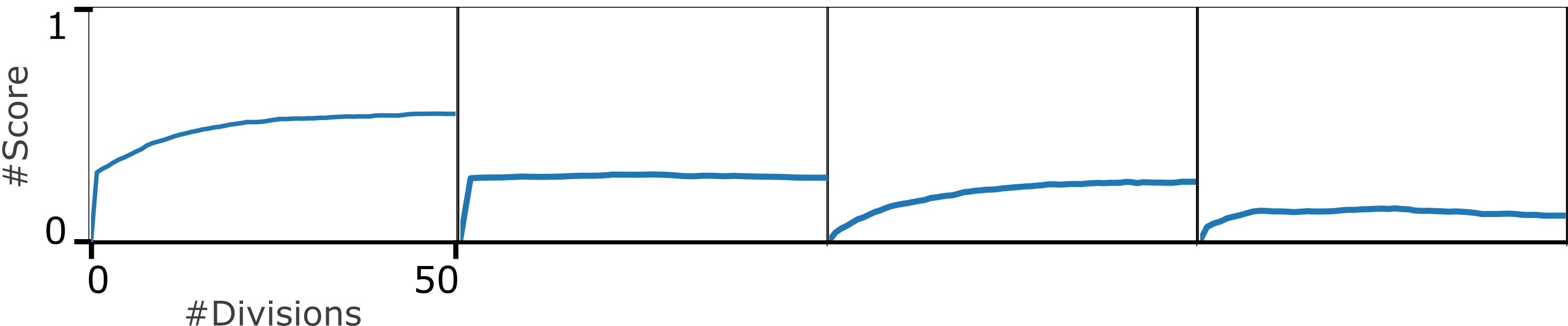

Supplement: Supplementary file 1 — Supplementary Information. [file 41598_2024_51386_MOESM1_ESM.zip › SupplementaryMaterial_NajmeKhorasani/2-img/Figure S2.pdf]
